# ROS-mediated abiotic stress-induced programmed cell death in plants

**DOI:** 10.3389/fpls.2015.00069

**Published:** 2015-02-18

**Authors:** Veselin Petrov, Jacques Hille, Bernd Mueller-Roeber, Tsanko S. Gechev

**Affiliations:** ^1^Institute of Molecular Biology and Biotechnology, PlovdivBulgaria; ^2^Department of Pharmacy, Faculty of Mathematics and Natural Sciences, University of Groningen, GroningenNetherlands; ^3^Department of Molecular Biology, Institute of Biochemistry and Biology, University of Potsdam, Potsdam-GolmGermany

**Keywords:** abiotic stress, programmed cell death, reactive oxygen species, signal transduction, stress adaptation

## Abstract

During the course of their ontogenesis plants are continuously exposed to a large variety of abiotic stress factors which can damage tissues and jeopardize the survival of the organism unless properly countered. While animals can simply escape and thus evade stressors, plants as sessile organisms have developed complex strategies to withstand them. When the intensity of a detrimental factor is high, one of the defense programs employed by plants is the induction of programmed cell death (PCD). This is an active, genetically controlled process which is initiated to isolate and remove damaged tissues thereby ensuring the survival of the organism. The mechanism of PCD induction usually includes an increase in the levels of reactive oxygen species (ROS) which are utilized as mediators of the stress signal. Abiotic stress-induced PCD is not only a process of fundamental biological importance, but also of considerable interest to agricultural practice as it has the potential to significantly influence crop yield. Therefore, numerous scientific enterprises have focused on elucidating the mechanisms leading to and controlling PCD in response to adverse conditions in plants. This knowledge may help develop novel strategies to obtain more resilient crop varieties with improved tolerance and enhanced productivity. The aim of the present review is to summarize the recent advances in research on ROS-induced PCD related to abiotic stress and the role of the organelles in the process.

## INTRODUCTION

All organisms need to cope with numerous stress factors throughout their lives by employing a wide variety of defensive strategies. While mobile individuals can in many cases just evade them, plants as sessile beings have developed sophisticated morphological, biochemical, and physiological adaptations in order to survive the detrimental effects of the environment (Hirayama and Shinozaki, 2010). To ensure proper and appropriate responses they have evolved sensitive detection systems and robust signal transduction mechanisms. Moreover, the different protective measures are triggered in a highly specific and tightly controlled manner so that adequate reactions are guaranteed without an excessive waste of resources.

**FIGURE 1:**
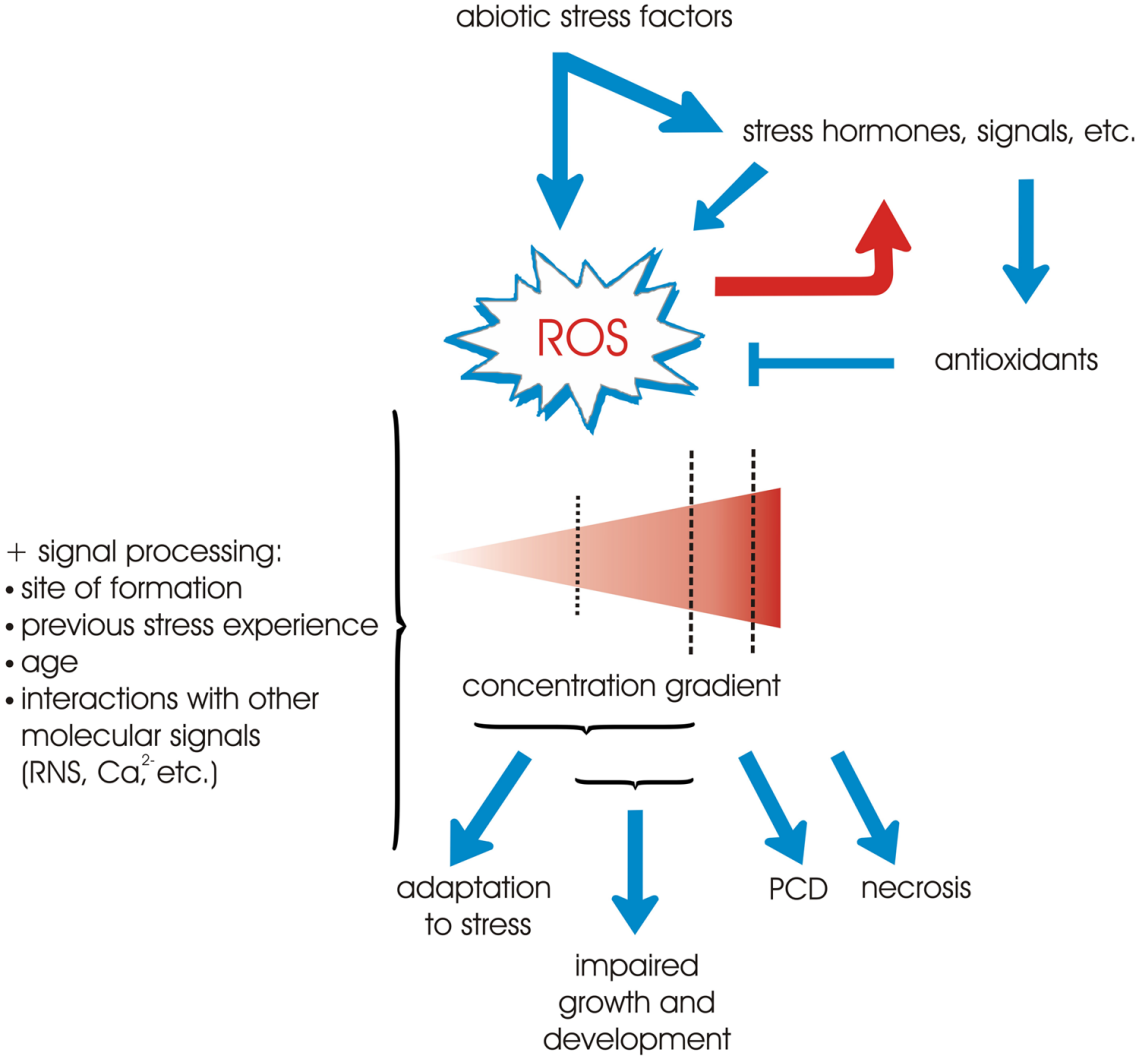
**An overview of the central role of ROS in the responses towards abiotic stress factors.** Different abiotic cues either directly or indirectly (through the action of other signals and hormones) lead to the production of ROS. In turn, ROS may influence a variety of signal transduction systems, thus providing positive or negative feedback control mechanisms (red arrow). The function of the antioxidant machinery is to prevent dangerous elevations of ROS levels. The outcome of ROS signaling depends mainly on the ROS concentration, but other factors like the site of ROS synthesis, previous stress exposure, developmental stage, and interaction with other signals like reactive nitrogen species (RNS) and Ca^2+^ are also integrated into the response. In general relatively weak stressors cause only a slight rise in ROS quantities which leads to adaptation. At more intensive abiotic stimuli the price for adaptation may be impaired growth and development of the plant. Severe stress usually causes massive accumulation of ROS and the initiation of PCD, or in extreme cases even necrosis of the tissue.

Plants face two principle types of stresses, biotic and abiotic, both of which can be deleterious. A wide spectrum of bacteria, fungi, and viruses attack plants at various stages of their development and the severity of the biotic stress may be altered by abiotic stress when it acts in parallel on the plant (Bostock et al., 2014). The latter includes extreme temperatures, high salinity, excessive light, water deprivation, pollutants such as ozone and herbicides, high concentrations of heavy metals, excessive UV radiation, and others (Gill and Tuteja, 2010). A common feature of the plants’ responses towards all these stressors is the production of the so called reactive oxygen species (ROS; Jaspers and Kangasjarvi, 2010). These are formed by the incomplete reduction (hydrogen peroxide – H_2_O_2_; superoxide radical – $O_{2}^{•-}$; hydroxyl radical – HO^∙^, etc.) or excitation (singlet oxygen – ^1^O_2_) of molecular oxygen (Gechev et al., 2006) and are strong oxidizers that can react with and damage a large variety of biological molecules (Petrov and Van Breusegem, 2012). They are regarded as byproducts of the aerobic way of life and are generated in different cellular compartments like chloroplasts, mitochondria and peroxisomes (Apel and Hirt, 2004). Despite their potential toxicity, ROS actually have a dual role *in vivo* depending on their concentration, site and duration of action, previous exposures to stress, etc. (Miller et al., 2010). In general, lower doses of ROS are employed as signals that mediate at least part of the responses towards stress while at higher concentrations they pose a significant threat that may eventually lead to programmed cell death (PCD; Gechev and Hille, 2005; **Figure 1**).

Programmed cell death is an active, genetically controlled process in which cells are selectively eliminated in a highly coordinated, multi-step fashion through the involvement of specific proteases and nucleases. Thus, only cells that are destined to die are destroyed and no damage to the neighboring cells is inflicted. Notably, PCD is central to a number of processes in non-stress conditions like the differentiation of tracheary elements, the formation of trichomes and root aerenchyma, abscission of floral organs, embryo formation, shaping the morphology of certain leaves, and many others (Gadjev et al., 2008). The balance between PCD and cell proliferation/elongation determines the growth rate of plant tissues (Van Breusegem and Dat, 2006). Hence, PCD is vital for normal growth and development of plants. On the other hand, PCD can be a consequence of severe abiotic and biotic stresses. Stress-induced PCD significantly affects plant yield and productivity and is therefore of fundamental importance to agriculture (Mittler and Blumwald, 2010). The changing climate conditions, combined with the expanding World’s population and the limited potential to increase the area of arable land, has driven both fundamental and applied research on stress responses and stress-induced PCD with the ultimate goal to minimize yield losses caused by environmental stresses (Cominelli et al., 2013; Rosenzweig et al., 2014). The aim of the present review is to summarize the recent advances made in the field of abiotic-stress induced PCD and especially the roles of ROS as signaling mediators in this phenomenon. Genes involved in these processes are listed in **Table 1**; we refer to many of them in the following text.

**Table 1:** Proteins that participate in the control of PCD processes provoked by different abiotic stresses.

Name	Function/activity	Related abiotic stresses	Reference
VPE, vacuolar processing enzyme	A PCD-executing protease	Salt, osmotic, heat, ER-stress	Li et al. (2012), Mendes et al. (2013), Kim et al. (2014)
BCL -2, B-cell lymphoma 2	Suppressor of VPE	Salt, osmotic stress	Kim et al. (2014)
AtCYSa and AtCYSb, cystatins	Cysteine protease inhibitors	Salt, drought, cold, oxidative stress	Zhang et al. (2008)
AtBOI, Botrytis susceptible1 interactor	Ubiquitinates BOS1, a transcription factor (TF) implicated in stress responses	Salt stress	Luo et al. (2010)
AtHSFA2, heat shock factor A2	Alleviates oxidative stress during heat shock	Heat stress	Zhang et al. (2009a)
mtHSP70, 70 kDa heat shock protein (HSP)	Maintains ΔΨm	Heat stress	Qi et al., 2011
AtKOD, kiss of death	A peptide that induces PCD responses upstream of mitochondrial dysfunction	Heat, oxidative stress	Blanvillain et al. (2011)
AtMPK6, MAP kinase 6	Upregulates VPE, part of the UVR8-independent pathway, drives responses to Cd^2+^	Heat, high UV, Cd^2+^-stress	Gonzalez Besteiro et al. (2011), Li et al. (2012), Jin et al. (2013)
AtLSD1, lesion simulating disease resistance 1	Negative regulator of PCD, interacts with catalases	Cold, UV-C, high-light stress	Dietrich et al. (1997), Muhlenbock et al., (2008), Huang et al. (2010), Wituszynska et al. (2014),
AtDEAR1, DREB and EAR motif protein 1	Transcriptional repressor of DREB	Cold stress	Tsutsui et al. (2009)
AtMPK3, MAP kinase 3	Involved in the UVR8-independent stress pathway	UV stress	Gonzalez Besteiro et al. (2011)
AtMC8, metacaspase 8	A part of the PCD pathway provoked by UV-radiation	UV-stress	He et al. (2008)
AtEDS1, enhanced disease susceptibility 1	Controls processes that extinguish excess energy, triacylglycerol lipase	UV-C, high light stress	Muhlenbock et al. (2008), Wituszynska et al. (2014)
AtPAD4, phytoalexin deficient 4	Regulator in PCD pathway controlled by LSD1, triacylglycerol lipase	High-light stress	Wiermer et al. (2005)
AtPARP, poly(ADP-ribose) polymerase	Synthesizes ADP-ribose polymers, using NAD^+^ as a substrate	High-light, drought, heat stress	De Block et al. (2005)
AtNTL -4, TF	Enhances ROS production during drought-induced senescence and heat stress	Drought, heat stress	Lee et al. (2012, 2014)
AOX1a, alternative oxidase 1a	Mitigates mitochondrial ROS burst	Al stress	Liu et al. (2014)
AtEXECUTER 1 and 2	Drives effects after ^1^O_2_ accumulation	^1^O_2_ stress	Lee et al. (2007)
AtSOLDAT, singlet oxygen-linked death activator	Suppresses ^1^O_2_-induced cell death	^1^O_2_ stress	Coll et al. (2009), Meskauskiene et al. (2009)
AtANAC013, TF	Conveys information about mitochondrial dysfunction	Mitochondrial stress	De Clercq et al. (2013)
AtbZIP28, TF	Transduces the UPR-response; part of the heat shock tolerance pathway	Heat, ER stress	Liu et al. (2007), Gao et al. (2008a)
AtbZIP60, TF	Transduces the UPR-response; important for salt stress tolerance	Salt, ER stress	Iwata and Koizumi (2005), Fujita et al. (2007)
AtNAC089, TF	Induces ER-triggered PCD	ER stress	Yang et al. (2014)
GmNAC30 and GmNAC81, TFs	Switch on VPE expression after ER and osmotic stress	Osmotic, ER stress	Mendes et al. (2013)
BI-1, Bax-inhibitor 1	Antiapoptotic protein in the ER	ER stress	Watanabe and Lam (2006), Isbat et al. (2009)
AtBiP2, ER luminal binding protein 2	ER chaperone protein, negative regulator of PCD	Cd^2+^ stress	Xu et al. (2013)
AtTCTP, translationally controlled tumor protein	Inhibits PCD progression, probably by sequestering Ca^2+^	Cold, salt, drought, Al stress	Hoepflinger et al. (2013)
AtUCP1, uncoupling protein 1	Uncouples e^-^ transport from ATP synthesis	Salt, drought stress	Begcy et al. (2011)
ZmABP9, ABRE binding protein 9	Controls ABA signaling and ROS accumulation	Drought, salt, cold stress	Zhang et al. (2011)

## ROS-INDUCED PCD AGAINST PARTICULAR ABIOTIC STIMULI

Plants respond to specific abiotic stresses in a manner that depends on the identity of the stress factor. Although some of the mechanisms, like the induction of ROS production (Jaspers and Kangasjarvi, 2010), are common and overlap between different kinds of abiotic cues (and even biotic stresses), some of the utilized signal transduction networks, recruited transcription factors (TFs), and modulated genes are specific for each kind of stress. Moreover, recent evidence shows that when subjected to a combination of multiple stresses, plants respond differently than when experiencing only a single type of stress (Mittler and Blumwald, 2010; Atkinson and Urwin, 2012). The study of reactions to multiple stresses will in the future be of particular interest, since in the field crops are often confronted by a combination of detrimental abiotic factors.

### DROUGHT STRESS-INDUCED PCD

Drought is among the abiotic factors that most strongly affect plant productivity and cause serious losses to crop yield (Godfray et al., 2010; Tester and Langridge, 2010). During water deprivation energy supply is severely restricted and ROS balance is impaired (Baena-Gonzalez et al., 2007; Miller et al., 2010). Not surprisingly, in the field drought usually occurs in combination with heat and their simultaneous action has a synergistic detrimental effect on plant productivity (Rampino et al., 2012). Prolonged drought stress is often associated with ROS accumulation, mainly due to decreased CO_2_ fixation concomitant with increased electron leakage to triplet oxygen which may eventually lead to PCD (Gechev et al., 2012). Fully functional ROS detoxifying systems are therefore of essential importance for the tolerance of plants towards drought and desiccation (Kranner et al., 2002). Therefore one of the main strategies to restrict ROS propagation and avoid unnecessary PCD under drought is to reduce ROS formation by downregulating chlorophyll synthesis and other components of the photosynthetic machinery (Farrant et al., 2007). Another factor that contributes to proper protection in these conditions is a well-balanced sucrose metabolism (Liu et al., 2013b). Sucrose fuels the glycolytic pathway which in turn feeds the oxidative pentose phosphate pathway. The latter produces reducing power (NADPH) which is needed for the synthesis of many non-enzymatic antioxidants including glutathione, ascorbic acid and flavonoids that are able to scavenge ROS (Bolouri-Moghaddam et al., 2010). In support of this hypothesis comes the finding that in two different cultivars of soybean the activities of glucose-6-phosphate dehydrogenase, a central enzyme of the pentose phosphate pathway, correlate with activities of the antioxidant enzymes glutathione reductase (GR), moodehydroascorbate reductase (MDHAR), and dehydroascorbate reductase (DHAR; Liu et al., 2013a). Sucrose as well as raffinose family oligosaccharides, which accumulate during dehydration, can also act as compatible solutes to protect from drought stress (Farrant et al., 2007). In addition, galactinol and raffinose can directly protect plants from oxidative stress as shown in *Arabidopsis* plants overexpressing galactinol synthase where elevated levels of galactinol and raffinose correlated with an increased tolerance to paraquat and abiotic stress (Nishizawa et al., 2008). Furthermore, both galactinol and raffinose can scavenge HO^∙^ *in vitro* (Nishizawa et al., 2008).

An important physiological adaptation during drought is the induction of leaf senescence. This process is executed by ROS-triggered PCD in selected leaves which allows nutrient remobilization and an overall reduction of the transpiratory surface. The phytohormones abscisic acid (ABA) and cytokinins also contribute to this phenomenon (Munné-Bosch and Alegre, 2004). Root tip meristems may also be affected by PCD as indicated by the degradation of organelles, an increased size of the vacuole and plasmalemma collapse during dehydration in *Arabidopsis thaliana* (Duan et al., 2010). This is an adaptive mechanism that is switched on to promote the development of lateral roots with enhanced stress tolerance which are important for post-stress recovery.

A prominent problem that occurs during periods of water deficit and other abiotic stresses is microspore abortion resulting in male sterility, which is due to the extreme sensitivity of the male reproductive program to adverse environmental conditions. This may be attributed to the increase in ROS concentrations following periods of drought and the subsequent PCD of the microspores (De Storme and Geelen, 2014). For example, when rice anthers are exposed to a short-term drought treatment accumulation of H_2_O_2_ is observed, accompanied by a reduced level of transcripts of antioxidant enzymes as well as ATP depletion. All these changes ultimately lead to PCD in developing anthers as confirmed by DNA fragmentation in these tissues (Nguyen et al., 2009).

Endogenous ABA, which regulates stomatal closure and thus CO_2_ uptake, rapidly accumulates during periods of water deprivation (Osakabe et al., 2014). The signaling pathway stimulated by ABA in guard cells at first leads to a boost of ROS production, which in turn provokes an increase in the cytosolic Ca^2+^ concentration. Subsequently, activation of anion and K^+^ eﬄux from the cell through specific ion channels results in the reduction of the guard cell turgor and thereby stomatal closure (Kwak et al., 2003). This process, however, is an example of a beneficial function of ROS which does not necessarily culminate in PCD. Another drought stress response mechanism in which ABA is implicated is the reorientation of polyamine (PA) metabolism in certain species like grapevine. In this case ABA induces PA accumulation and extrusion to the apoplast, where the PAs are then targeted by apoplastic amine oxidases. One of the products of the following reaction is H_2_O_2_, which may orchestrate further stress responses or induce PCD when present in high concentrations (Toumi et al., 2010).

### SALT/OSMOTIC-STRESS INDUCED PCD

This kind of stress increases ROS concentrations and possibly leads to PCD by different mechanisms. In C3 plants the main source of ROS is the accelerated rate of photorespiration, which contributes to up to 70% of the accumulated H_2_O_2_ (Noctor et al., 2002). In C4 and CAM plants photorespiration proceeds in a negligible pace, which makes the chloroplast electron transport chain the main ROS producer. Moreover, during osmotic stress NADPH oxidases and apoplastic diamine and PA oxidases are activated as is mitochondrial respiration, which further elevates ROS concentration (Abogadallah, 2010). NADPH oxidase-produced superoxide anions are necessary for the execution of a PCD program switched on by both NaCl and sorbitol in tobacco cells (Monetti et al., 2014). However, the molecular reactions following the ionic NaCl and non-ionic sorbitol treatments do not completely overlap (Monetti et al., 2014) which suggests that these responses are highly specific. PA oxidases contribute directly to the H_2_O_2_ load in the apoplast since they catabolize PAs like spermidine, which are extruded into the apoplasts under high salinity, and produce H_2_O_2_ as a byproduct (Moschou et al., 2008). Salinity-provoked PCD is not restricted to higher plants, as demonstrated by Affenzeller et al. (2009), who report this phenomenon in the freshwater green alga *Micrasterias denticulata.*

An important feature of salt-induced PCD is that it is strongly affected by ion disequilibrium and may be the result of invasion of Na^+^ into the cytosol accompanied by a deficiency of K^+^ (Kim et al., 2014). The eﬄux of K^+^ is influenced by hydroxyl radicals and in turn regulates different enzymes involved in PCD (Demidchik et al., 2010). These findings suggest that maintenance of ionic homeostasis and proper Na^+^/K^+^ ratios could be used to manipulate salt stress tolerance in crops (Huh et al., 2002; Teakle and Tyerman, 2010). For example, a recent study in rice demonstrated that BCL-2, an antiapoptotic protein, can regulate vacuolar processing enzymes (VPE), which play a crucial role in many PCD processes, by modulating ion fluxes. Overexpression of BCL-2 significantly reduces NaCl-induced K^+^ eﬄux, represses the expression of VPEs and thus alleviates PCD symptoms (Kim et al., 2014). A detailed model of the ion-specific signaling that triggers PCD under high Na^+^ concentrations has been proposed by Shabala (2009). Another mode of modulating the stress and PCD responses is through cysteine protease inhibitors (cystatins). For instance, the overexpression of two *Arabidopsis* cystatins – AtCYSa and AtCYSb – was shown to improve the tolerance not only to salt stress, but also to drought, cold and oxidation (Zhang et al., 2008).

Mitochondria play a pivotal role in salt-induced PCD in plants, especially in non-photosynthetic cells. The reason is that in these tissues mitochondria are the main ROS-producing compartment. The ROS burst in mitochondria may lead to a loss of outer membrane potential, which is an early marker for PCD in *A. thaliana* (Yao et al., 2004). Indeed, in tobacco protoplasts subjected to salt stress, the PCD program is mediated by ROS and depends on the opening of the mitochondrial permeability transition pores (PTP; Lin et al., 2006).

Since excessive ROS production switches on the PCD program and may jeopardize the survival of the plant, many of the responses towards abiotic stresses, including high salt concentrations, aim at minimizing the ROS threat. This is usually achieved by a very tight control of the antioxidant systems and upregulation of different antioxidants, both enzymatic and non-enzymatic. A good example is the halophytic shrub *Prosopis strombulifera* which after treatment with Na_2_SO_4_ boosts antioxidant activities, the synthesis of flavonoid compounds and increases the carotenoid/chlorophyll ratio (Reginato et al., 2014). Another mechanism to keep ROS levels under control, when subjected to salt stress, is the accumulation of compatible solutes. These organic osmolytes are needed to keep proper osmotic potential in stressed tissues but contribute also by alleviating oxidative stress (Liu et al., 2011; Duan et al., 2012). Interestingly, recent experiments performed with barley and wheat demonstrated that the rise in compatible solute concentrations provides cross-tolerance also to UV-B stress (Puniran-Hartley et al., 2014). Thus, despite being metabolically expensive in terms of required energy and carbon input, the salt-induced *de novo* synthesis of compatible solutes may serve multiple purposes and improve the chances of survival in hostile environments which pose a combination of abiotic challenges. The integrity of antioxidant systems is of vital importance for seeds as well. Their ability to germinate depends on the ROS status and a compromised antioxidant machinery, especially in combination with abiotic stresses, may significantly reduce germination efficiency. Such is the case in *A. thaliana* seeds which lack the cytosolic antioxidant ASCORBATE PEROXIDASE 6 when subjected to salt and heat stress (Chen et al., 2014).

### EXTREME TEMPERATURE-INDUCED PCD

High temperatures significantly affect plant growth and development by triggering massive metabolic, physiological, and genetic reprogramming. Like other abiotic stress factors, heat shock is known to induce PCD (Vacca et al., 2004). Tobacco cells treated with high temperatures produce drastically larger quantities of ROS. These are a prerequisite for a successful PCD since addition of the antioxidants ascorbate or superoxide dismutase (SOD) to the cultures supports cell survival. Moreover, the authors show that both H_2_O_2_ and $O_{2}^{•-}$ are required and the absence of either of them compromises the PCD process. Mitochondria also play an important role in these conditions. They release functionally active cytochrome c in a ROS-dependent manner, which contributes to the activation of caspase-like proteases in the cytosol that execute the PCD program (Vacca et al., 2006). A newer study demonstrates that PCD is triggered only when the temperature rises above a specific threshold which is associated with metabolic changes that are considerably different to those in response to less intensive stimuli (Marsoni et al., 2010). For example, only in severe heat shock-treated cells is a down regulation of antioxidant proteins observed. This in turn leads to the perturbation of the redox homeostasis and plays a role in the PCD events. When subjected to shorter or less intensive heat stimuli plants can become primed and thus more resistant to subsequent stressful events. This enhanced thermo-tolerance is achieved by an improved antioxidant capacity, which in wheat seedlings is based on upregulated expression and higher activities of SODs, GR, and peroxidases (Wang et al., 2014) and once again confirms the importance of ROS homeostasis for plant performance under these conditions. An interesting fact is that proline, which acts as a compatible solute, ROS detoxifier and regulator of the redox balance (Szabados and Savoure, 2010), is inhibitory to *Arabidopsis* seedlings during heat stress (Lv et al., 2011). This is in contrast to the role of proline for other types of stresses, especially salt/osmotic stress, where it has a protective role (Verbruggen and Hermans, 2008). These facts support the notion that every type of abiotic influence triggers a specific sequence of adaptive reactions.

An important detail is that the type and localization of ROS may influence the execution of the PCD program that follows. Recently it was observed that the exogenous application of the unspecific antioxidants ascorbate and glutathione to heat-treated *A. thaliana* cells actually favors the initiation of PCD, probably because cellular stress levels are reduced and the uncontrolled necrotic death is avoided. In contrast, treatment with catalase, which is specific only for H_2_O_2_, temporarily suppresses PCD. The authors hypothesize that it is the H_2_O_2_ which may function as the PCD-inducing mobile signal, while the other types of ROS and the products of their activity serve as positive or negative regulators of the components of the PCD machinery (Doyle and McCabe, 2010).

It has been proposed that heat shock transcription factors (HSFs), which are central to the coordinated expression of heat shock proteins (HSPs) and other stress-related genes, have an important role in the ROS-mediated response to heat stress because they may function as ROS-sensitive sensors (Miller and Mittler, 2006). Both, the *HSF* and *HSP* genes are highly induced by ROS (Gechev et al., 2005; Piterkova et al., 2013). Lack of HSFA2 causes accumulation of higher ROS levels, more severe mitochondrial dysfunction and a lower survival rate due to increased PCD (Zhang et al., 2009a). In rice, overexpression of the mitochondrial HSP mtHSP70 may suppress the PCD response at elevated temperatures and after H_2_O_2_ treatment by maintaining the mitochondrial transmembrane potential (ΔΨm) and reducing ROS amplification (Qi et al., 2011). Other components of the molecular machinery transducing the heat shock signal include the mitogen-activated protein kinase 6 (MPK6) and γVPE (Li et al., 2012). According to this study, the cascade that is triggered at elevated temperatures starts with the accumulation of ROS and an increase of the cytosolic Ca^2+^ concentration. Calcium in turn is bound by a form of the ubiquitous second messenger calmodulin (CaM3) which leads to the activation of MPK6. Finally, MPK6 upregulates *γVPE* expression and γVPE precursors are targeted to the vacuole, where they are activated to process a number of vacuolar hydrolases and proteases associated with PCD.

Among the major sites of ROS production during heat shock is the photosynthetic apparatus. As a consequence of high temperature the photosystem II complexes (PSII) may be severely damaged due to cofactor dissociation. This has numerous effects, one of which is the formation of ROS (Mathur et al., 2014). Elevated concentrations of H_2_O_2_ may also be generated in peroxisomes due to photorespiratory activity which is significantly accelerated at elevated temperatures. The reason is the reduction of the relative specificity of ribulose-1,5-bisphosphate carboxylase/oxygenase (RuBisCO) for CO_2_ compared to O_2_ and the lower relative solubility of CO_2_ than O_2_ (Jordan and Ogren, 1984). However, the shift of H_2_O_2_ production from chloroplasts to peroxisomes due to photorespiration can be regarded as less dangerous since peroxisomes are better suited to cope with the H_2_O_2_ load mainly due to the abundance of the reductant-independent enzyme catalase (Wingler et al., 2000).

Low temperature (both chilling and freezing) on its own can also induce PCD in plants (Koukalova et al., 1997; Lyubushkina et al., 2014). Tobacco BY-2 suspension cells progressively develop characteristic features of PCD, including DNA condensation and fragmentation (laddering), after exposure to 5–6^∘^C for two to 5 weeks (Koukalova et al., 1997). Interestingly, 11% of the cells did not die even after prolonged incubation at low temperature. Winter wheat (*Triticum aestivum* L.) cell cultures exposed to freezing treatment (-8^∘^C) undergo PCD accompanied by ROS accumulation, DNA fragmentation, and the release of cytochrome c from mitochondria into the cytosol (Lyubushkina et al., 2014). LESION SIMULATING DISEASE RESISTANCE 1 (LSD1) is a plant-specific negative regulator of PCD and its mutation causes ROS accumulation and runaway cell death (Jabs et al., 1996; Dietrich et al., 1997). LSD1 interacts with catalases in *A. thaliana* to keep ROS under control and loss of its function renders plants more susceptible to low temperature-induced cell death (Huang et al., 2010; Li et al., 2013).

Low temperature is particularly damaging when combined with elevated light intensities. During such conditions, the photosynthetic electron transport chains are overreduced, there is increased generation of ROS, and the antioxidant system cannot cope with it. As a result, photoinhibition or/and cell death occurs (Murata et al., 2007).

### UV LIGHT-INDUCED PCD

The UV spectrum comprises three ranges of different wavelengths: UV-A (315–390 nm), UV-B (280–315 nm) and UV-C (below 280 nm). Of these, UV-C is often used as a reliable trigger of PCD in scientific studies (Gao et al., 2008b) despite the fact that in natural conditions UV-C is absorbed by the ozone layer in the stratosphere and is therefore of little physiological relevance. UV-B exposure, in turn, has been widely considered as a potent stressor of plants in the past, but in the last years this concept has been reconsidered (Hideg et al., 2013) since many studies failed to demonstrate direct evidence for UV-B provoked stress in terrestrial ecosystems (Ballare et al., 2011). It was proposed that low, ecologically relevant UV-B doses induce so called “eustress,” a condition that primes the plant for future oxidative pressure but does not impede growth and development *per se*, while high UV-B intensity leads to a state of “distress” which is associated with a ROS burst and may eventually result in PCD (Hideg et al., 2013). In experimental conditions the UV-B irradiation that is used is usually strong enough to overload the antioxidant capacities of the cells and cause oxidative stress.

Strong UV light has been shown to be deleterious to both animals and plants. In animals it causes apoptosis (Kulms and Schwarz, 2000) while in plants it leads to apoptotic-like symptoms like DNA laddering and fragmentation of the nucleus (Danon and Gallois, 1998). This abiotic cue leads to a sharp rise in $O_{2}^{•-}$ concentrations in chloroplasts due to e^-^ excitation by energy transition in different photosensitizers (Hideg et al., 2002). The plastidic SODs can transform $O_{2}^{•-}$ to H_2_O_2_ which is another consequence of UV stress. In *Rosa X damascena* (the Damask rose) the peak of H_2_O_2_ accumulation is 60–90 min after irradiation (Murphy and Huerta, 1990). Recently, it was shown that in UV-B irradiated tobacco leaves the antioxidant activities targeting H_2_O_2_ are the most activated (Majer et al., 2014). This suggests that H_2_O_2_ scavenging would be crucial for survival in such conditions. Interestingly, it was shown by experiments with fluorescent sensors of different ROS forms that the relative portion of ^1^O_2_ formed as a result of UV treatment is low in comparison to the other ROS (Hideg et al., 2002). To prevent PCD, high UV-B doses trigger a defense program aiming to alleviate the symptoms of oxidative stress including synthesis of the stress hormones salicylic acid (SA), jasmonic acid (JA), and ethylene, upregulation of pathogenesis-related proteins and genes participating in senescence processes (Bandurska et al., 2013). Stress avoidance and stress tolerance strategies induce morphological changes and adaptations of the metabolism to favor the accumulation of flavonoids in epidermal cells, lignin deposition in cell walls, and to reinforce the antioxidant system with both enzymatic (SOD, catalase, peroxidases) and non-enzymatic ROS scavengers (carotenoids, tocopherols, ascorbic acid, PAs, etc.; Ueda and Nakamura, 2011; Zhang et al., 2012).

Two independent UV-B response pathways were proposed to act in accordance to the UV-B fluence rate (Nawkar et al., 2013). The UV RESISTANCE LOCUS8 protein (UVR8), characterized as a major UV-B photoreceptor (Kliebenstein et al., 2002; Rizzini et al., 2011), mediates photomorphogenic adaptive responses to moderate levels of UV-B. To achieve this UVR8 interacts with the CONSTITUTIVE PHOTOMORPHOGENESIS 1 (COP1) protein and induces the TFs ELONGATED HYPOCOTYL (HY5) and HY5 HOMOLOG (HYH; Brown and Jenkins, 2008). In contrast, high fluence rates trigger a stress UVR8-independent pathway that includes a mitogen-activated protein kinase (MAPK) cascade involving the antagonistic action of MAP KINASE PHOSPHATASE 1 (MKP1) and the MAP-kinases MPK3 and MPK6 (Gonzalez Besteiro et al., 2011).

The LSD1 and ENHANCED DISEASE SUSCEPTIBILITY 1 (EDS1) proteins are antagonistic regulators of the acclimation to UV-C stress, since *lsd1* mutants are characterized by enhanced cell death after UV-C treatment, while *eds1* plants manifest the opposite phenotype. They exert their function by controlling the processes that extinguish excessive energy and modulate ROS homeostasis (Wituszynska et al., 2014). In mitochondria, which are affected by UV-C, a ROS burst leads to a decline in the ΔΨm. Other symptoms of the mitochondrial dysfunction include changes of their distribution and mobility (Gao et al., 2008b). The rise of ROS quantities and the damage to mitochondria are important for the normal completion of a PCD program as indicated by the fact that treatment with the antioxidant ascorbic acid or the inhibitor of the mitochondrial PTPs, *i.e.* cyclosporine, reduces the rate of PCD. In summary, ROS that mediate the signal for PCD after UV-C treatment mainly originate in mitochondria and chloroplasts as described above, which in normal physiological conditions are in close proximity to allow metabolic interchange (Logan and Leaver, 2000). Moreover, light is also necessary for this type of PCD to occur as well as caspase-like activities as demonstrated by the ability of caspase inhibitors to impede the process (Danon et al., 2004; Zhang et al., 2009b).

### HIGH LIGHT-INDUCED PCD

Light with intensity higher than 1000 μmol photons m^-2^s^-1^ can be very damaging to plants and especially to chloroplasts (Szechynska-Hebda and Karpinski, 2013). In these conditions, leading to excess excitation energy (EEE), leaves are exposed to more energy than they can actually utilize in photosynthesis or dissipate without being harmed (Wilhelm and Selmar, 2011). The enzymatic processes of CO_2_ fixation are slowed, which is accompanied by the synthesis of higher than necessary concentrations of NADPH and ATP. The increase in reducing equivalents reduces the plastoquinone pool and/or inhibits the oxygen evolution complex responsible for the photolysis of water. As a result the PSII complexes are inactivated, a process known as photoinhibition, which goes hand in hand with photoreduction of molecular oxygen to ROS (Anderson et al., 1997). If the antioxidant systems fail to keep ROS propagation under control, growth retardation and PCD may occur (Mateo et al., 2004). Examples of the damage that ROS can cause in these conditions include the degradation of glutamine synthetase, phosphoglycolate phosphatase, and the large subunit of RuBisCO as well as the increased occurrence of carbonyl groups in stromal proteins (Stieger and Feller, 1997).

Plants react with a complex adaptive systemic acquired acclimation response (SAA) towards EEE. Recently it has been shown that SAA and PCD signals are regulated by stomatal conductance, photorespiration, ROS homeostasis, hormonal balance as well as local changes in the plasma membrane electric potential called photoelectrophysiological signaling (PEPS; Szechynska-Hebda et al., 2010). The propagation of EEE-induced PCD initiated by changes in the redox state of the chloroplasts is dependent on the activities of several regulatory genes like *LSD1*, *EDS1*, *PHYTOALEXIN DEFICIENT 4* (*PAD4*) and *ETHYLENE INSENSITIVE2* (*EIN2*), whose protein products function upstream of ROS production (Muhlenbock et al., 2008; Karpinski et al., 2013).

Plants with compromised antioxidant systems or proper composition of the photosynthetic machinery have been widely used to study the PCD responses triggered by massive ROS accumulation provoked by excessive light. For example, experiments with the *Arabidopsis* mutants *vte1npq1*, deficient in α-tocopherol and zeaxanthin, and *ch1*, lacking chlorophyll *b*, have demonstrated that the rise of the ^1^O_2_ concentrations following high light treatment can directly lead to PCD due to the cytotoxicity of ^1^O_2_ (Triantaphylides et al., 2008). Interestingly, the *ch1* mutant can be rescued from this ^1^O_2_-dependent PCD if subjected to mild light stress acclimation first (Ramel et al., 2013). However, in another mutant, *npq1lut2*, which is unable to produce the ^1^O_2_ quenchers lutein and zeaxanthin, ^1^O_2_ responses did not include PCD (Alboresi et al., 2011), suggesting that the identity of ^1^O_2_-induced reactions under high light depends on the source of ^1^O_2_ in the chloroplasts and the intensity of the signal. H_2_O_2_ has also been shown to initiate PCD in excessively light-stressed plants (Vandenabeele et al., 2003; Mullineaux et al., 2006).

### HEAVY METAL-INDUCED PCD

It is well-known that high concentrations of heavy metal ions can stimulate a ROS burst (Dat et al., 2000; Cho and Seo, 2005). Evidence for this is the increased lipid peroxidation in plants subjected to heavy metal stress. For example, after treatment of *Solanum nigrum* with cadmium (Cd), malondialdehyde accumulates, indicating augmented oxidation of membrane lipids by ROS (Deng et al., 2010). One should also keep in mind that some heavy metals, like Fe and Cu, may participate in the Haber–Weiss reaction which leads to the production of the hyper-active hydroxyl radical (HO^∙-^; Kehrer, 2000).

Many reports study PCD induced by different heavy metal ions. Cd, for example, appears to trigger PCD by a pathway that involves the endoplasmatic reticulum (ER), in which unfolded proteins accumulate (Xu et al., 2013). This has been demonstrated in *Nicotiana tabacum* BY-2 cells in which markers for ER stress (*NtBLP4* and *NtPDI*) were upregulated after exposure to Cd, while chemical and protein (expression of the *Arabidopsis AtBiP2* gene) chaperones can suppress this induction, as well as the induction of the PCD-related gene *NtHsr203J*. Ethylene, which is modulated by ROS formation, also participates in the reactions of plants to Cd stress since it stimulates the onset of PCD (Yakimova et al., 2006; Liu et al., 2008). It appears that the cellular response to Cd involves complex crosstalk between ROS, NO, Ca^2+^ ions, and hormones. In a study with pea plants it was shown that one of the primary effects of Cd^2+^ treatment is actually depletion of cytosolic Ca^2+^ which negatively regulates components of the antioxidant system like SOD. This results in elevated H_2_O_2_ and $O_{2}^{•-}$ concentrations. On the other hand, the reduction of Ca^2+^ affects also NO production, leading to its depression. But the decrease in NO can in turn promote the accumulation of $O_{2}^{•-}$, further exacerbating the oxidative load. The lipid peroxidation in membranes caused by ROS turns on JA and ethylene mediated pathways (Rodriguez-Serrano et al., 2009). Many genes undergo expression changes in such conditions, including elements of MAPK cascades as well as enzymes involved in ethylene, NO and PA metabolism (Kovalchuk et al., 2005; Chmielowska-Bak et al., 2013). The relationship between Cd and oxidative stresses manifests also in experiments with *Arabidopsis* mutants which are characterized by tolerance to both of them. Such is the case for plants overexpressing an aldehyde dehydrogenase (*ALDH3*; Sunkar et al., 2003), mutants of a MAP-kinase (*mpk6*; Jin et al., 2013) and the *atr* lines which are less sensitive to Cd- and paraquat-induced oxidative stress (Radeva et al., 2010).

Aluminum (Al) is another highly toxic element which causes damage to both animals, including humans, and plants. Al negatively affects the soil-derived water and nutrient supply in plants by inhibiting the division and elongation of root tip cells (Samac and Tesfaye, 2003). ROS burst, mitochondrial dysfunction, and induction of PCD are considered to be the main symptoms of exposure to Al at the cellular level (Li and Xing, 2010). The sequence of events in these conditions includes stimulation of ROS synthesis, for example by a dramatic upregulation of the $O_{2}^{•-}$ producer respiratory burst oxidase homolog (*Rboh*), opening of the mitochondrial PTP, reduced potential of the inner mitochondrial membrane, release of cytochrome c into the cytosol and finally – an activation of the caspase-like 3 protease (Huang et al., 2014). Interestingly, the PCD features are relieved after the application of protease and human caspase inhibitors, which suggests involvement of caspase-like proteases in the cell death process. A similar cell death-suppression effect is observed after addition of antioxidants like catalase, ascorbic acid and its precursor L-galactonic acid-γ-lactone as well as compounds known to interact with and inhibit NADPH oxidases (Yakimova et al., 2007), which once again confirms the importance of ROS for Al-induced PCD. The mitochondrial enzyme alternative oxidase (AOX), which is specific for plants and serves to prevent over-reduction of the mitochondrial electron transport chain, is also involved in Al stress as demonstrated recently (Liu et al., 2014). According to this study, *AOX1a* upregulation is provoked by Al-derived $O_{2}^{•-}$ in the mitochondria and AOX1a in turn can mitigate the following PCD by maintaining proper mitochondrial functions.

Tungsten (W) is not as abundant as Al and Cd, but its quantities can be augmented as a result of human activities. Like other heavy metals, W is also harmful, with a negative impact on plant growth caused by binding to and inactivation of molybdoenzymes and switching on PCD. The cell death initiated by high W concentrations has been studied in pea root cells and according to the results the mechanism involves the ER, similar to Cd (Adamakis et al., 2011). A possible explanation for the effect of the inhibitory role of W on molybdoenzymes and the activation of ROS-dependent PCD came from the observation that the *xdh1* mutant of *A. thaliana*, which is deficient for the molybdenum-containing enzyme xanthine dehydrogenase, accumulated xanthine and exhibited symptoms of premature senescence (Brychkova et al., 2008). Furthermore, the *xdh1* mutant subjected to darkness and subsequent re-exposure to light accumulated ROS and exhibited cell death to a larger extent than the wild type, an effect that could be attenuated by the addition of allantoin or allantoate, indicating that these metabolites protect cells from cellular damage by ROS (Brychkova et al., 2008).

Unlike the non-essential metals like Al, W, and Cd mentioned above, some other metallic elements, such as zinc (Zn), are important micronutrients with an array of functions. Although Zn deficiency has many negative consequences to plants, including growth retardation and reduction of the efficiency of the antioxidant system (Sharma et al., 2004), accumulation of Zn is highly deleterious (Wang et al., 2009). The mechanisms of Zn toxicity have not been fully elucidated yet, but similar to other heavy metals the symptoms involve a ROS imbalance and ultimately may lead to PCD. In a study with *Solanum nigrum* roots it was demonstrated that Zn^2+^ initially boosts NO production and NO in turn negatively regulates components of the antioxidant system thereby increasing ROS concentrations and triggering PCD. The rise of NO was proven to be necessary for this Zn-induced PCD since its inhibition prevented ROS accumulation and the subsequent cell death (Xu et al., 2010). Interestingly, Zn participates also in beneficial PCD processes throughout plant development. In Norway spruce (*Picea abies*) somatic embryos Zn manifests an anti-cell death effect and is contained (in physiological concentrations) within the proliferating cells, while those that need to be subjected to PCD are Zn-depleted (Helmersson et al., 2008). All these findings confirm that in the case of Zn the dose makes the poison and disruption of its homeostasis in either direction - depletion or accumulation – can have dramatic consequences to plants.

### FLOODING/LOW OXYGEN-INDUCED PCD

Flooding represents a global problem which seriously diminishes crop yield. It has been estimated that nowadays more than 17 million km^2^ of arable land are prone to flooding and in certain areas the risk of flooding may further increase in the future (Shabala et al., 2014). The main problem to plants in these conditions is the significantly reduced availability of molecular oxygen which causes hypo- or anoxia. To alleviate the symptoms related to hypo/anoxia, plants resort to a defense strategy which involves the formation of specific air channels in the submerged tissues called aerenchyma. These interconnected cellular spaces facilitate gas diffusion and reduce the metabolic requirements of the flooded organs (Lenochova et al., 2009). Aerenchyma is formed after cells are selectively eliminated by a PCD process that shares some similarities with apoptosis and cytoplasmic cell death in animals (Gunawardena et al., 2001).

Numerous lines of evidence support the notion that ethylene and ROS are both integral parts of the mechanism of aerenchyma development. For example, Steffens et al. (2011) demonstrated that exogenous application of H_2_O_2_ stimulates lysigenous aerenchyma emergence in rice stems. Moreover, one of the highly upregulated genes during waterlogging in maize is *Rboh*, which, as mentioned earlier, is a major ROS producer (Rajhi et al., 2011). In addition, accompanying mitochondrial dysfunction and the accumulation of metal ions during flooding also increase ROS concentration (Shabala et al., 2014). On the other hand, it is long known that ethylene synthesis is enhanced in a low-oxygen environment and subsequently ethylene triggers PCD (He et al., 1996a,b). In a recent study it was shown that treatment of wheat seedlings with the precursor of ethylene, i.e., 1-aminocyclopropanecarboxylic acid (ACC), promotes aerenchyma formation similar to H_2_O_2_ but the addition of the NADPH oxidase inhibitor diphenyleneiodonium chloride (DPI) suppresses the effect of ACC (Yamauchi et al., 2014). Applying DPI alone prevents the spread of submergence-dependent aerenchyma formation (Parlanti et al., 2011). These findings support the model that ROS are essential for aerenchyma development and participate in the transduction of an ethylene signal from which they act downstream. A similar mechanism involving ethylene and ROS operates in epidermal cells which need to undergo PCD in order to allow the emergence of adventitious roots in rice as a response to flooding (Steffens et al., 2012).

As in other cases of abiotic stress-induced PCD, the reactive nitrogen species (RNS) NO may also play a role during exposure to low oxygen levels. NO is mainly produced in mitochondria, mostly by the reduction of nitrite by the electron transport chain (Gupta and Igamberdiev, 2011). Importantly, a negative feedback loop between ROS, NO, and AOX was found to exist in mitochondria; NO is able to actually induce *AOX* expression, probably to reduce the ROS and NO burden (Fu et al., 2010). Although this mechanism is supposed to keep ROS and NO concentrations under control, it may be overrun by ROS and NO from non-mitochondrial origin in which case PCD, caused by the ROS and NO accumulation, may occur (Gupta et al., 2012). During hypoxia another small moleculare, i.e., H_2_S, antagonizes ROS effects. In experiments with pea seedlings, treatment with H_2_S significantly lowered root tip death by mitigating membrane damage by ROS and suppressing ethylene production. In contrast, the death of root tip cells was promoted after application of inhibitors of endogenous H_2_S synthesis (Cheng et al., 2013).

## INTEGRATION OF PCD SIGNALS FROM THE ER, CHLOROPLASTS, AND MITOCHONDRIA

As already described in the previous chapter, signals from different types of abiotic cues are often generated in specific cellular compartments like chloroplasts, mitochondria, or the ER. These organelles are prone to stress but in the same time possess mechanisms to process the information in accordance to the intensity of the stressor as well as the developmental stage and the previous experience of the plant. The local metabolic imbalances and ROS accumulation caused by the harmful abiotic conditions produce signals which are integrated and relayed to other parts of the cell, including the nucleus, and as a result cellular physiology and biochemistry are readjusted (Suzuki et al., 2012) to allow the plant to adapt. However, should the damage caused by the stressor be insurmountable, a PCD program may be induced.

### CHLOROPLASTS, MITOCHONDRIA, AND THEIR ROLE IN PCD

As the organelles in which photosynthesis occurs, chloroplasts are extremely sensitive to abiotic factors, especially those related to light and UV irradiation. Due to the intensive energy fluxes, constant electron transport, and the demand for reducing power they are among the major sites of ROS production in plant cells (Baier and Dietz, 2005). Information of the current redox-status of these organelles, which reflects also the ROS concentrations, may be transmitted by monitoring the state of the plastoquinone, ascorbate, and glutathione pools (Foyer and Noctor, 2009). Chloroplasts, as well as mitochondria, send signals to the nucleus, a process known as retrograde signaling (Suzuki et al., 2012), and in this way they may influence the expression of numerous genes in accordance with their redox condition, including triggers of PCD.

A well-studied example is the ability of ^1^O_2_ to initiate suicide responses. ^1^O_2_ is generated mainly by excited triplet state chlorophyll, photosensitizers like protochlorophyllide in the presence of light or in the PSII light harvesting complex when the electron transport chain is over-reduced as a result of stress (Apel and Hirt, 2004). To achieve ^1^O_2_ accumulation, scientists have often applied a dark-to-light shift of the conditional *flu* mutant of *Arabidopsis*, which retains high levels of the chlorophyll precursor and photosensitizer protochlorophyllide (op den Camp et al., 2003; Laloi et al., 2007). Work with this mutant has provided evidence that ^1^O_2_ does not induce PCD merely due to its cytotoxic effects, but actually switches on a genetically controlled program driven by factors like EXECUTER1 and EXECUTER2 (Lee et al., 2007). The light-dependent release of ^1^O_2_ alone cannot provoke cell death, but needs in addition the blue light-specific photoreceptor cryptochrome (Danon et al., 2006). Suppressor mutants of the *flu* mutation called *singlet oxygen-linked death activators* (*soldat*), in which the ^1^O_2_-induced cell death is eliminated, have also been found (Coll et al., 2009; Meskauskiene et al., 2009). A ^1^O_2_-provoked PCD is also observed in the *oep16* mutant, which is defective in NADPH:protochlorophyllide oxidoreductase A import and accumulates protochlorophyllide (Samol et al., 2011). Another way of ^1^O_2_ accumulation is observed in the previously mentioned *chlorina (ch1)* mutant, which is deficient in chlorophyll *b* (Ramel et al., 2013). The ^1^O_2_-induced gene expression in *chl* resembles *flu.* Moderately elevated light intensities can repress the jasmonate pathway and induce acclimation, while jasmonate signaling is strongly induced by high light intensities that trigger PCD (Ramel et al., 2013).

Another ROS species, i.e., hydrogen peroxide, appears to antagonize the effects of ^1^O_2_ in chloroplasts (Laloi et al., 2007) which supports the idea that the identity of the ROS signal is of primary importance for the ensuing outcome. In another study it was found that in *Arabidopsis* cell suspension cultures only the treatment with the ^1^O_2_-producing chemical Rose Bengal (RB), but not with H_2_O_2,_ triggers PCD, again confirming the importance of the nature of the ROS signal (Gutierrez et al., 2014). Moreover, these authors show that the ^1^O_2_-mediated cell death program requires functional chloroplasts since dark-incubated cells, which lack them, had the PCD response suppressed. *Arabidopsis* cell suspension cultures were used to investigate also the apoptotic-like PCD (AL-PCD) that follows a heat shock treatment at 55^∘^C (Doyle et al., 2010). However, in this case the presence of viable chloroplasts seemed to reduce the occurrence of AL-PCD at the expense of necrotic cell death. In addition, treatment of chloroplast-containing cells with antioxidants increased the proportion of AL-PCD and decreased the number of necrotic cells which demonstrates the crucial role of plastid-originated ROS for PCD regulation. A possible explanation of these observations could be that cultures containing chloroplasts, which are major ROS producing sites, more easily lose control of their ROS metabolism after the heat shock, which prevents the genetically organized PCD process and leads to the more chaotic necrosis instead.

As mentioned earlier, mitochondria are active participants in abiotic stress-induced PCD. The decrease of ΔΨm, most often accompanied by the release of cytochrome c, are common early markers of plant PCD as they are in animals (Yao et al., 2004). This in turn results in the oxidation of a number of macromolecules, including lipids. The oxidation of cardiolipin negatively influences the strength of cytochrome c binding to the inner mitochondrial membrane and leads to its diffusion into the intramembrane space. At the same time, the oxidative stress causes permeabilization of the transition pores in the outer membrane thus letting cyt c pass into the cytosol (Ott et al., 2007). In plants there is no evidence that cyt c can directly activate PCD (Reape and McCabe, 2008), but it appears to be one of the prerequisites, together with ROS (Vacca et al., 2006). It has been proposed that when the PTP are open and cyt c is dissipated, even more ROS are generated in the mitochondrial electron transport chain (mtETC), which forms a positive feedback loop to amplify the original signal (Reape and McCabe, 2008). Therefore, the cyt c leakage seems to mark the “point of no return” after which PCD execution is inevitable. Similarly to chloroplasts, mitochondria can also produce retrograde signals to notify the nucleus of their current stress status. An example is the TF ANAC013 in *Arabidopsis* which was recently described to convey information about mitochondrial dysfunction to the nucleus and to lead to increased oxidative stress tolerance. Interestingly, ANAC013 is located in the ER under non-stress conditions, which demonstrates the tight physical and functional interactions between the ER and the mitochondria (De Clercq et al., 2013).

### THE ER AND ITS ROLE IN PCD

The ER is an organelle with essential functions for protein sorting, post-translational modifications (such as *N*-glycosylation), and proper folding (Csala et al., 2006). In case these processes are impaired due to detrimental environmental factors, a specific phenomenon termed as “ER stress” can ensue, which is characterized by the accumulation of misfolded proteins (Cai et al., 2014). Adaptation to ER stress is achieved through the so called unfolded protein response (UPR), in the course of which signals from the ER are sent to the nucleus, which in turn increases the protein folding capacity of the cell (Watanabe and Lam, 2009). TFs like CabZIP1 from pepper and AtbZIP28 and AtbZIP60 from *Arabidopsis*, which participate in the transduction of the UPR, are already known to contribute to resistance to abiotic stressors like heat, drought, and high salinity (Fujita et al., 2007; Gao et al., 2008a; Moreno and Orellana, 2011). However, when the ER stress signal is too intense, a cell death program is triggered instead (Boyce and Yuan, 2006). Recently, the *Arabidopsis* membrane-associated TF ANAC089, which relocates to the nucleus under severe ER stress, was identified as one of the controllers that cause PCD in such conditions (Yang et al., 2014).

Similar to other varieties of stress-induced PCD, ROS take part in the ER-PCD as well, mostly by provoking the mobilization of Ca^2+^ from the ER to mitochondria, where Ca^2+^ opens the mitochondrial PTPs (Williams et al., 2014). This proves once more that the ER and mitochondria can exchange information and integrate common PCD signals. As in other cases of PCD triggered by adverse conditions, the VPE can be implicated in the execution of the cell death program during severe ER stress. In soybean (*Glycine max*), for example, VPE may be switched on by the cooperative action of two NAC TFs, i.e., GmNAC30 and GmNAC81, which together integrate both ER- and osmotic stress-induced cell death responses (Mendes et al., 2013).

Among the numerous PCD regulators in animals, much attention has been paid on the BCL2-associated X protein (BAX), as a pro-apoptotic factor, and on BAX inhibitor-1 (BI-1), as an anti-apoptotic protein (Isbat et al., 2009). No *BAX* homologs have been reported in plants, while *BI-1* is well conserved also in the plant kingdom. Its protein product is resident in the ER and takes an important part in the regulation of cell death (Watanabe and Lam, 2009). *BI-1* is upregulated in response to many adverse abiotic stimuli and contributes to plant tolerance to a variety of stress factors (Watanabe and Lam, 2006; Isbat et al., 2009). Interestingly, the overexpression of *Arabidopsis BI-1* (*AtBI-1*) efficiently prevents PCD driven by the exogenous application of H_2_O_2_ or ectopic expression of mammalian BAX, but does not influence the accompanying ROS accumulation (Kawai-Yamada et al., 2004). On the other hand, some antioxidant enzymes like Fe-SOD, APX, and glutathione *S*-transferase (GST) were identified as BAX inhibitors in a genetic screen with yeast (Watanabe and Lam, 2009), which implies that ROS are important components of BAX-mediated cell death. These findings suggest that BI-1 acts downstream of ROS in the PCD pathways in plants.

It was recently reported that an NADPH-dependent cytochrome P450 oxidoreductase is able to interact with human BI-1 and as a result the electron uncoupling between this P450 reductase and cytochrome P450 2E1, known to be a major source of ROS at the ER membrane, is reduced. This, in turn, leads to a decrease in ROS production that may lower the unfolded and misfolded protein accumulation in the ER (Ishikawa et al., 2011). However, whether such an adaptive mechanism, that alleviates ER stress by relaxation of the oxidative load, is operational in plants is currently unclear.

## INFLUENCE OF THE DEVELOPMENTAL STAGE ON THE SEVERITY OF OXIDATIVE STRESS DAMAGE AND SENESCENCE

As mentioned in the Introduction and **Figure 1**, the outcome of ROS accumulation depends not only on their concentration and site of production, but also on other factors like previous stress experience and interaction with other signals. The developmental stage of a plant is also of prime importance for its ability to withstand adverse environmental conditions or initiate senescence. Ethylene can accelerate the leaf senescence symptoms in wild-type *A. thaliana* plants older than 17 days but no visible yellowing is observed in younger plants (Jing et al., 2002). This ethylene-induced leaf senescence is regulated in part by the *ONSET OF LEAF DEATH (OLD)* genes (Jing et al., 2005). It has been demonstrated that from a certain age, the photosynthetic activity in chloroplasts is reduced, while the oxidative stress progressively increases (Munné-Bosch and Alegre, 2002), which further contributes to the aging process. This negative impact on the ROS balance is due to numerous metabolic alterations related to age, such as the reduction of the ascorbate levels and the activity of antioxidant enzymes (Khanna-Chopra, 2012). The age-dependence of the severity of abiotic stress was investigated in growing maize leaf cells (Kravchik and Bernstein, 2013). The authors show that the response to salinity is influenced by age since younger cells possess more potent ROS-scavenging mechanisms in comparison to their older counterparts. Similar results were reported in a study of the acclimation to water deficit (Sperdouli and Moustakas, 2014). Younger leaves of *A. thaliana* owned their better tolerance to moderate water deficit mainly through the increased protection from ROS, which includes also more pronounced sugar accumulation, more stimulated photochemical quenching and higher levels of proline. In an experiment involving treatment of different squash cultivars with the potent oxidative stress inducer paraquat, Yoon et al. (2011) demonstrated that 14 out of 18 cultivars are characterized by lower levels of paraquat damage in younger leaves. The significance of the developmental stage for the tolerance is not only true for abiotic factors, but for biotic stressors as well, like in the case of tobacco leaves which were differentially susceptible to the metabolic products of *Alternaria alternata* (Jia et al., 2010).

## CONCLUSION

Programmed cell death is a central process in plant development, but in addition is triggered by a diverse spectrum of abiotic (and biotic) stresses, as highlighted here. Although the molecular and biochemical mechanisms underlying abiotic stress-induced PCD are being unraveled, several important questions remain to be answered in the future, such as: Which of the genes, proteins and regulatory networks involved in developmental PCD are also recruited for abiotic stress-induced PCD? At which point do the upstream signaling pathways that activate developmental and stress-triggered PCD converge and to what extent determine (or restrict) developmental programs the entrance into abiotic stress-induced PCD? Which of these genes and pathways are conserved in the plant kingdom (crops included) and which of them are species-specific or restricted to particular taxonomic groups? How is abiotic stress-induced PCD integrated into whole-plant physiology and development (e.g., with respect to the initiation of new leaves after drought stress which may have caused damage and loss of older leaves)? Answering these and other important questions about the abiotic stress-induced ROS-dependent PCD can ultimately help to develop high-yielding and at the same time more resilient crop varieties.

## Conflict of Interest Statement

The authors declare that the research was conducted in the absence of any commercial or financial relationships that could be construed as a potential conflict of interest.
